# COVID-19 mortality among immigrants by duration of residence in Sweden: a population-based cohort study

**DOI:** 10.1177/14034948241244560

**Published:** 2024-04-10

**Authors:** Sol P. Juárez, Enrico Debiasi, Matthew Wallace, Sven Drefahl, Eleonora Mussino, Agneta Cederström, Mikael Rostila, Siddartha Aradhya

**Affiliations:** 1 Department of Public Health Sciences, Stockholm University, Sweden; 2 Centre for Health Equity Studies (CHESS), Stockholm University/Karolinska Institutet, Stockholm, Sweden; 3 Stockholm University Demography Unit (SUDA), Department of Sociology, Stockholm University, Sweden; 4 Aging Research Center (ARC), Karolinska Institutet, Stockholm, Sweden

**Keywords:** COVID-19, immigrants, vulnerability, susceptibility, Sweden

## Abstract

**Background:**

Explanations for the disproportional COVID-19 burden among immigrants relative to host-country natives include differential exposure to the virus and susceptibility due to poor health conditions. Prior to the pandemic, immigrants displayed deteriorating health with duration of residence that may be associated with increased susceptibility over time. The aim of this study was to compare immigrant–native COVID-19 mortality by immigrants’ duration of residence to examine the role of differential susceptibility.

**Methods:**

A population-based cohort study was conducted with individuals between 18 and 100 years old registered in Sweden between 1 January 2015 and 15 June 2022. Cox regression models were run to estimate hazard ratios (HRs) and 95% confidence intervals (CIs).

**Results:**

Inequalities in COVID-19 mortality between immigrants and the Swedish-born population in the working-age group were concentrated among those of non-Western origins and from Finland with more than 15 years in Sweden, while for those of retirement age, these groups showed higher COVID-19 mortality HRs regardless of duration of residence. Both age groups of immigrants from Africa and the Middle East showed consistently higher COVID-19 mortality HRs. For the working-age population: Africa: HR<15: 2.46, 95%CI: 1.78, 3.38; HR≥15: 1.49, 95%CI: 1.01, 2.19; and from the Middle East: HR<15: 1.20, 95%CI: 0.90, 1.60; HR≥15: 1.65, 95%CI: 1.32, 2.05. For the retirement-age population: Africa: HR<15: 3.94, 95%CI: 2.85, 5.44; HR≥15: 1.66, 95%CI: 1.32, 2.09; Middle East: HR<15: 3.27, 95%CI: 2.70, 3.97; HR≥15: 2.12, 95%CI: 1.91, 2.34.

**Conclusions:**

**Differential exposure, as opposed to differential susceptibility, likely accounted for the higher COVID-19 mortality observed among those origins who were disproportionately affected by the pandemic in Sweden.**

## Introduction

International migrants have been disproportionately affected by the COVID-19 pandemic, with higher risks of infection, hospitalisation, and death compared to their host-native counterparts [1-3]. Sweden was no exception, with immigrants, particularly those from low- to middle-income countries, identified as populations at risk from the beginning of the pandemic [4-8].

The higher COVID-19 burden among immigrants is usually discussed in the context of social inequalities in exposure to the virus or susceptibility, which refer to the possibility that immigrants are less equipped than natives to fight the disease due to, for example, a higher prevalence of pre-existing health conditions [9, 10]. Since previous research has shown that social factors, often associated with higher exposure to the virus (e.g. crowded housing), only partially account for the observed disadvantages, questions have been raised regarding the role of differential susceptibility [5]. However, empirical evaluations of susceptibility via pre-existing health conditions are challenging due to the lack of relevant information (e.g. data on smoking, alcohol consumption, or obesity) and the differential risk of underdiagnosis among immigrants [11, 12]. These challenges therefore call for alternative ways of examining this potential mechanism.

In this study, we draw from previous evidence that suggests that the health advantage that characterises newly arrived immigrants disappears with longer duration of residence. This phenomenon, which indicates increasing health vulnerability, leads to the expectation that immigrants with longer duration of residence will experience greater disadvantages in dealing with an infectious disease. This would result in a pattern where longer residency correlates with higher COVID-19 mortality rates. Though exploratory, a comparison between the pattern of duration of residence from all-cause mortality before the pandemic with the pattern of COVID-19 mortality during the pandemic will allow us to indirectly examine the differential susceptibility hypothesis without underreporting diagnoses or health behaviours.

Using register data, the aim of this study was to evaluate whether the excess COVID-19 mor-tality among immigrants relative to the Swedish-born population varied by immigrant duration of residence.

## Data and methods

This study is part of the COVIS project [13], approved by the Swedish Ethical Review Authority (Decision nos. 2022-00428-01 and 2021-05754-02).

### Data

A cohort study was conducted using data from multiple national administrative registers linked throu-gh pseudonymised personal identification numbers. The study population was identified from the Total Population Register (TPR), which acts as the base for the production of official population statistics in Sweden. From the TPR we identified the country of birth, birth year, and the year of arrival in Sweden (for migrants). Sociodemographic information (e.g. disposable income, education, and civil status) was retrieved from the Longitudinal Integration Database for Health Insurance and Labour Market Studies (LISA). We used the Death Register to determine the date of death and cause.

### Study population

**Figure 1.**
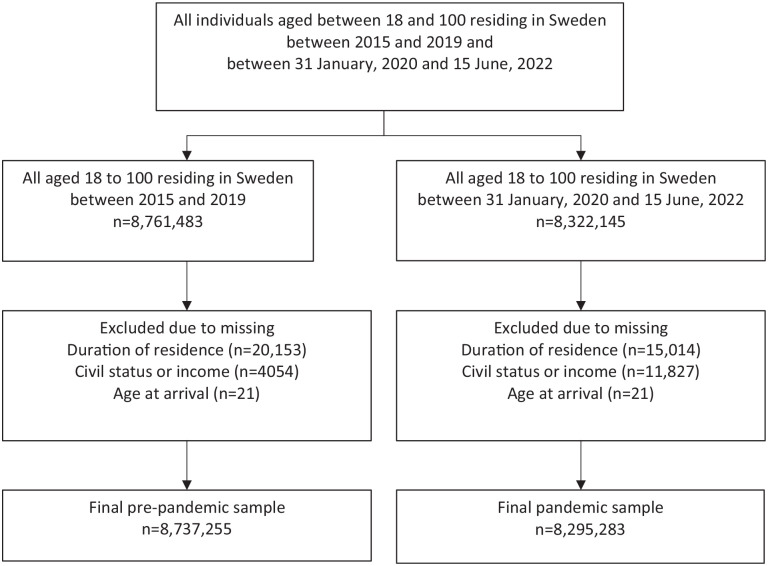
Selection chart and study population.

The study population was divided in two follow-up groups: (1) the “pre-pandemic” follow-up included all individuals between 18 and 100 years old residing in Sweden from 1 January 2015, their 18th birthday, or immigration, who were followed until death (outcome), emigration, their 100th birthday, or 31 December 2019 – whichever occurred first. (2) the “pandemic” follow-up was defined as starting on 31 January 2020 (beginning of the pandemic), their 18th birthday, or immigration, and followed until COVID-19 death (outcome), other cause of death, emigration, their 100th birthday, or 15 June 2022 – whichever occurred earliest. We excluded individuals from the two groups who were missing information on duration of residence, age at arrival or socioeconomic variables. The final population for the pre-pandemic period consisted of 8,737,255 individuals, while the population was 8,295,283 for the pandemic period (Figure 1)

### Exposure

Exposure was region of origin by duration of residence. We divided the study population into 11 groups based on the geographical location of the origin country: Sweden, Finland, other Nordic countries, Western countries (EU-28 countries excluding the Nordics, Great Britain and Northern Ireland, North America, Oceania), Eastern Europe (European countries not in the EU-28 or the Nordics, former Soviet Union, former Yugoslavia), Latin America, Africa, the Middle East, Southeast Asia, the rest of Asia, and MISS (i.e. individuals with missing country of birth). Individuals from each of these regions were sub-divided according to their duration of residence in Sweden into one of two categories: <15 years and ⩾15 years. We also compared immigrants and the Swedish population by sex using a more disaggregated categorisation of duration of residence in years: <5, 5–9, 10–14, 15–19, 20–24, 25–29, 30–34 and ≥35.

### Outcome

In the pre-pandemic period, we analysed all-cause mortality. During the pandemic period, we analysed COVID-19 deaths and all other causes of mortality excluding deaths related to COVID-19.

### Covariates

All models were adjusted for individuals’ sociodemographic characteristics, including age (baseline hazard), sex, income, education, civil status and place of residence to account for compositional differences. For income, we considered individual’s disposable income divided into quintiles calculated by sex, year and age group. Educational attainment was categorised into primary (lower than high school), secondary (high school level) and post-secondary (higher than high school) and MISS (i.e., individuals with missing education attainment). Civil status reflected being single, married or in a registered partnership, divorced or separated, or widowed. Lastly, we included county of residence fixed effects. All-covariates (except sex) vary annually. Unadjusted estimates are presented in the Appendix.

### Method

We used Cox proportional hazard models to estimate mortality hazard ratios (HRs) and 95% confidence intervals (95%CIs) for the different immigrant groups, using age as the underlying time scale. When considering cause-specific mortality (i.e. COVID-19, all other causes), models were run separately for each cause of death, and individuals dying from a different cause than the one considered were right censored. The analyses were run separately for the working-age (18–65) and retirement-age (66+) populations.

### Sensitivity analysis

We ran analyses restricting the working-age group to those who arrived in Sweden after the age of 18 in order to examine the impact of age at arrival on the results.

All analyses were performed using STATA version 15 [14].

## Results

The population studied during the pre-pandemic and the pandemic periods had similar characteristics (see Table I and Table A1 in the Appendix for descriptive information corresponding to the pre-pandemic pe-riod). Some differences by origin can be observed. Finns represented the oldest immigrant group on average, with more than 54% falling within the oldest age category. In contrast, immigrants from Africa, the Middle East, Southeast Asia and the rest of Asia were predominantly of working age. Specifically for the COVID period, a large share of Finns and immigrants from other Nordic countries had resided in Sweden for long periods, while people from Africa and Asia had resided in Sweden for periods shorter than 15 years at the time of the research. Most individuals were married or cohabiting, particularly among Middle Eastern and Asian populations. There were no marked differences in the distribution of educational attainment, yet some immigrant groups were skewed towards lower incomes, particularly those from Africa and the Middle East.

**Table I.** Descriptive statistics for the study population during the pandemic.

	Sweden	Finland	Rest of Nordics	Western	Eastern Europe	Latin America	Africa	Middle East	Southeast Asia	Rest of Asia	MISS
*N*	6,447,452	139,140	77,942	184,149	312,396	72,795	197,550	512,425	137,533	73,539	140,362
Deaths	191,011	8453	3046	3412	5408	731	1143	4093	607	326	2049
COVID deaths	15,088	881	240	333	583	108	193	778	76	61	222
All deaths excl. COVID	175,923	7572	2806	3079	4825	623	950	3315	531	265	1827
Sex, %											
Men	50	39	49	53	48	48	53	56	39	45	52
Women	50	61	51	47	52	52	47	44	61	55	48
Age group, %											
16–45	41	11	32	56	52	52	70	63	70	71	52
46–65	31	34	34	27	31	34	25	30	26	24	35
66–100	28	54	34	17	17	14	5	7	4	5	14
Age on arrival, %											
<18		39	28	18	21	36	23	23	24	15	28
⩾18		61	72	83	79	64	77	77	76	85	72
Duration of residence, %											
<15		8	29	58	48	30	80	61	60	70	36
⩾15		92	71	42	52	70	29	39	40	30	64
Civil status, %											
Not married	43	26	31	42	28	39	35	30	35	32	33
Married or Reg. Par.	39	41	43	43	49	37	43	53	50	55	51
Divorced	12	21	18	12	18	22	20	15	13	12	13
Widow(er)	6	12	8	4	4	2	2	3	2	2	4
Income quintiles, %											
1	16	19	23	33	29	28	34	37	36	41	30
2	19	21	16	16	21	22	27	27	20	19	19
3	21	23	17	16	20	19	19	17	17	15	17
4	22	21	19	16	17	16	12	11	13	12	17
5	23	17	24	19	14	15	8	8	14	13	18
Educational attainment, %											
Primary	16	27	17	10	14	14	31	29	21	12	11
Secondary	46	43	34	25	38	39	35	32	27	20	35
Tertiary	38	28	34	49	36	42	24	31	41	54	45
MISS	1	2	15	16	12	5	9	9	11	13	10

*Note*: MISS: missing; Reg. Par.: registered partnership. Total may not always sum up to 100% due to rounding.

The descriptive information corresponding to the pre-pandemic period can be found in Table A1 in the Appendix.

### Pre-pandemic mortality

**Figure 2.**
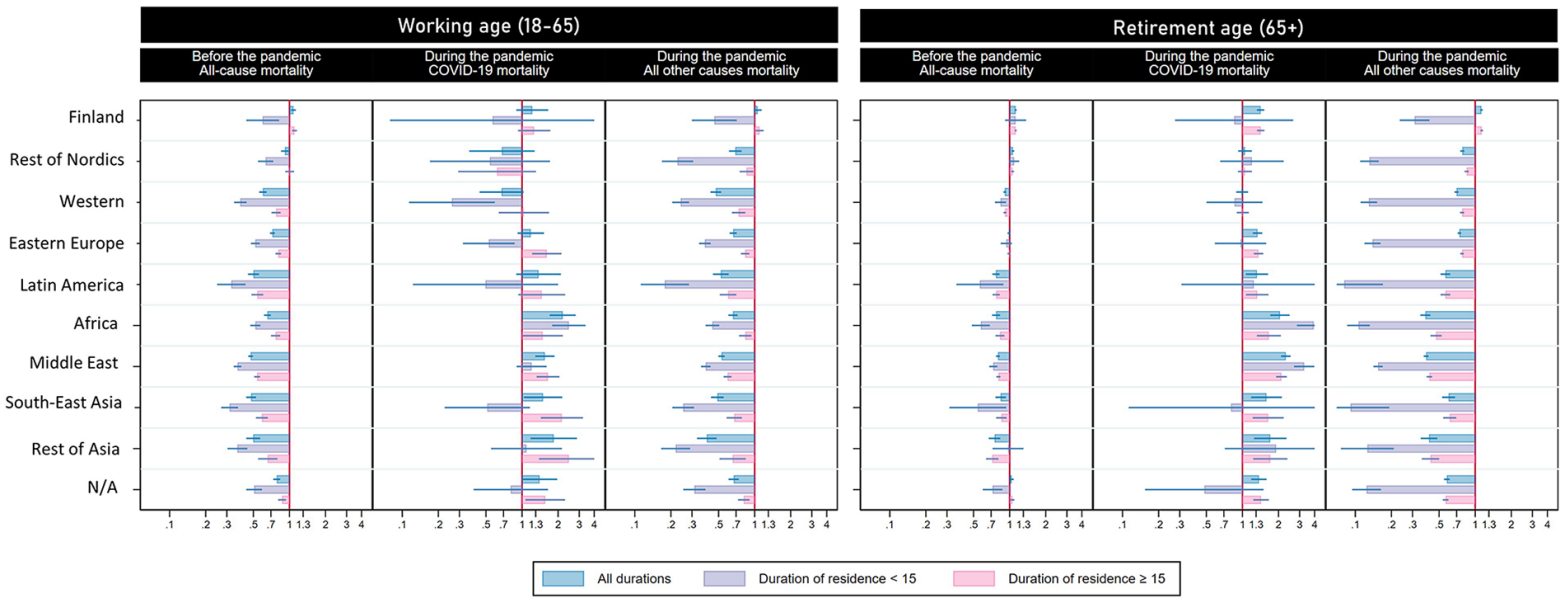
All-cause mortality for the pre-pandemic period (2015–2019) and COVID-19 mortality and all other causes of death during the pandemic by immigrant origin and duration of residence (<15 and ≥15 years). *Note*: Models adjusted for age, sex, civil status, income, education and county of residence.

**Figure 3.**
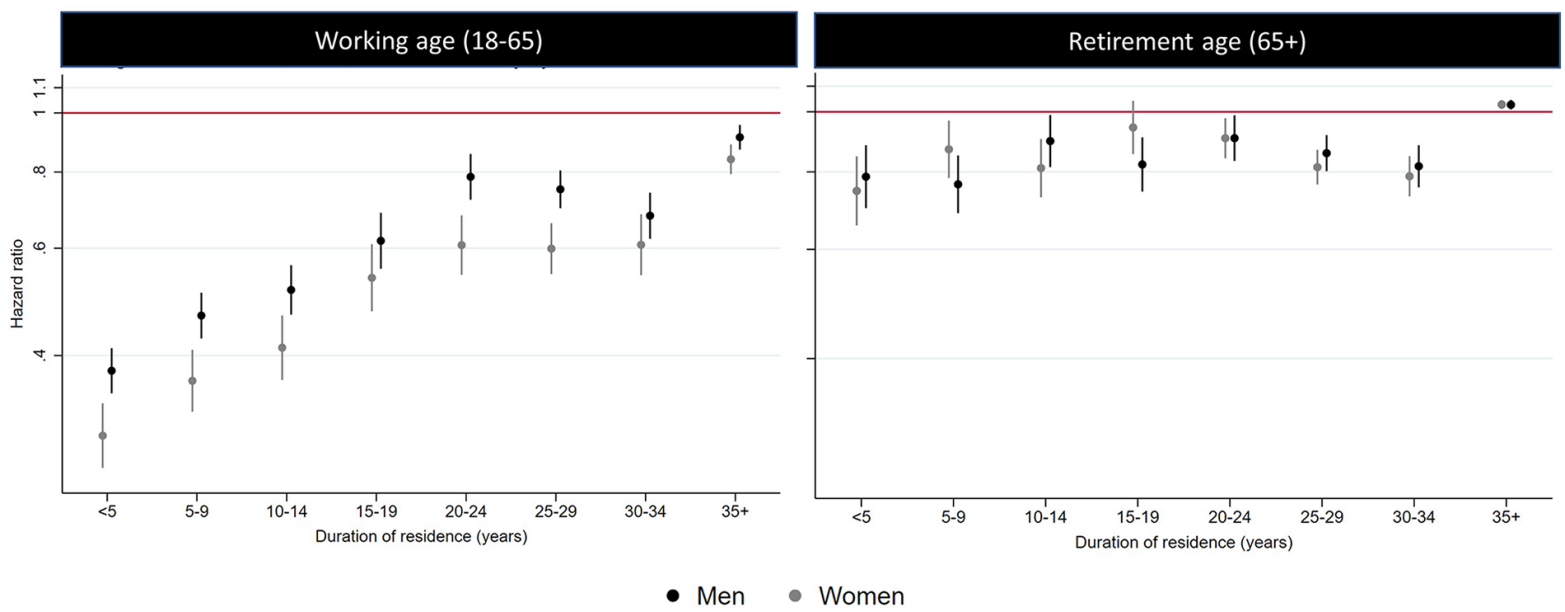
All-cause mortality for pre-pandemic period (2015–2019) by sex and duration of residence. *Note*: Models adjusted for age, civil status, income, education and county of residence. Unadjusted models are presented in the Appendix.

The pre-pandemic mortality advantage was observed in all immigrant origins (except for Finland) among the working-age population, with significant variation by duration of residence. For all origins, the mortality advantage was more prominently observed among those who had spent less than 15 years in Sweden. Mortality hazards for those residing in Sweden for over 15 years approached, but never reached, the levels of the Swedish-born reference group. Exceptions were observed for the rest of Nordics group, which fully converged with the Swedish-born population and Finland. The levels of the mortality advantage as well as the pattern of convergence with a higher duration of residence were less clear for the retirement group, except for those of non-Western origins (i.e. Latin America, Africa, Middle East, Southeast Asia and, to lesser extent, the rest of Asia) (see Figure 2. Estimates can be found in Tables A2 and A3 in the Appendix). When combining the immigrant origins to be able to expand the category of duration of residence, a linear association between the mortality hazards by duration of residence was observed for both men and women during the first 15 years of residence. However, the pattern tended to be constant or even reversed after 15 years (see Figure 3. Estimates can be found in Tables A4 and A5 of the Appendix).

### COVID-19 mortality

Most immigrant origins in Sweden experienced higher COVID-19 mortality hazards compared to the Swedish-born population, regardless of whether they were of working or retirement age (Africa, Middle East, rest of Asia, Southeast Asia, Latin America Eastern Europe and Finland). This pattern contrasted with the general mortality advantage observed for all origins (excluding Finland) before the pandemic for all causes of death, and during the pandemic for all other causes. In contrast, immigrants from the ‘rest of Nordics’ and ‘Western’ groups showed similar or lower COVID-19 mortality hazards compared to the Swedish-born population (Figure 2. Estimates can be found in Tables A6–A9 in the Appendix,).

COVID-19 mortality hazards varied by duration of residence but the pattern differed for most origins between the working- and retirement-age groups. For the working-age population, COVID-19 mortality hazards were generally higher among individuals who had lived in Sweden for more than 15 years, compared to both the Swedish-born population and those who had lived in the country for less than 15 years. This was consistent for all origins except for rest of Nordics, who showed lower hazards compared to the Swedish-born population regardless of duration of residence (HR_<15_ 0.54, 95%CI: 0.17, 1.71; HR_⩾15_ 0.62, 95%CI: 0.29, 1.31); and for Africa, which showed higher COVID-19 hazards among those who had spent less (as opposed to more) than 15 years in Sweden compared to the Swedish-born population (HR_<15_ 2.46, 95%CI: 1.78, 3.38; HR_⩾15_ 1.49, 95%CI: 1.01, 2.19). Surprisingly, for most origins, the COVID-19 mortality hazards were lower than the Swedish-born population among those who had lived in Sweden for less than 15 years. This was the case for immigrants from Western countries (HR_<15_ 0.26, 95%CI: 0.11, 0.59), Eastern Europe (HR_<15_ 0.53, 95%CI: 0.32, 0.87) and, with a lower level of precision, from Southeast Asia (HR_<15_ 0.51, 95%CI: 0.23, 1.16), Latin America (HR_<15_ 0.50, 95%CI: 0.12,2.00), rest of Nordics (HR_<15_ 0.54, 95%CI: 0.17, 1.71) and Finland (HR_<15_ 0.57, 95%CI: 0.08, 4.08).

For the retirement-age group the results were less striking. On the one hand, compared to the Swedish-born population, the lower COVID-19 hazard among immigrants with less than 15 years of residence was less prominent. On the other hand, apart from Africa (HR_<15_ 3.94, 95%CI: 2.85, 5.44; HR_⩾15_1.66, 95%CI: 1.32, 2.09), other origins showed the highest COVID-19 hazard among those who had resided in Sweden less (as opposed to more) than 15 years: Middle East (HR_<15_ 3.27, 95%CI: 2.70, 3.97; HR_⩾15_ 2.12, 95%CI: 1.91, 2.34) and with less statistical accuracy also the rest of Asia (HR_<15_ 1.91, 95%CI: 0.71, 5.12; HR_⩾15_ 1.71, 95%CI: 1.23, 2.37).

For both the working- and retirement-age populations, immigrants from Africa and the Middle East showed higher COVID-19 mortality hazards compared to the Swedish-born population regardless of their duration of residence. In the case of immigrants from Africa, the highest COVID-19 mortality hazards were observed in the group that had spent less than 15 years in Sweden. For the Middle East, this was true only among the retirement-age group since the COVID-19 mortality hazard for the working-age population with less than 15 years in Sweden was substantially lower than for those who had spent 15 years or more in the country.

The results from the sensitivity analysis restricting the population to adult immigrants (i.e. excluding those who were under the age of 18 on arrival) were consistent with the main models (see Table A10 in the Appendix).

## Discussion

Our findings showed that immigrants from the Mid-dle East, Africa, South Asia, the rest of Asia, Finland, and to a lesser extent from Latin America and Eastern Europe, experienced higher COVID-19 mortality compared to the Swedish-born population. Our study contributes to previous research showing that, among the working-age population, higher COVID-19 mortality is mainly concentrated among immigrants residing in Sweden for 15 years or more. In fact, for most immigrants in the working-age population, living in Sweden for less than 15 years was as-sociated with lower COVID-19 mortality hazards compared to the Swedish-born population. Ex-ceptions included African and Middle Eastern immigrants, who experienced higher COVID-19 mortality than Swedes regardless of duration of residence or age group, a pattern that may explain the disproportionately higher COVID-19 mortality experienced by these groups during the pandemic. Moreover, the risk of COVID-19 mortality among African immigrants was notably higher among those with a shorter (as opposed to longer) duration of residence regardless of the age group. For the retirement-age population specifically, differences by duration of residence were smaller and the patterns were somewhat different than for the younger group. This was clearest among immigrants from Africa, the Middle East and the rest of Asia, which compared to the Swedish-born population, had higher COVID-19 mortality hazards with a shorter (as opposed to longer) duration of residence. Sub-analyses on immigrants who arrived as adults suggested that our findings were not driven by age on arrival rather than duration of residence.

Our pre-pandemic results confirmed the mortality advantage and a health deterioration with increasing duration of residence across immigrants of all origins among the working-age population, as well as among those of non-Western origins for the retirement-age group. This is the first study that has shown this pattern in Sweden, which supports our hypothesis that susceptibility increases with duration of residence, potentially influencing the levels of COVID-19 mortality. Further studies should determine the mechanisms underlying this pattern in Sweden; this could include considering the role of acculturative stress and cumulative disadvantage, and the exposure of inequality including, but not limited to, racism and discrimination [15-18].

Immigrants from most origins maintained a mortality advantage before the pandemic despite living in Sweden for over 15 years, indicating that exposure rather than susceptibility likely played a key role in the higher COVID-19 mortality rates among immigrants. This is further supported by the observation that immigrants from some origins faced higher COVID-19 mortality hazards regardless of their duration of residence, with those residing for less than 15 years sometimes experiencing even higher rates. In fact, immigrants from the origins that showed the largest disadvantage during the pandemic (i.e. Africa and the Middle East) are overrepresent-ed in socioeconomically disadvantaged and more densely populated residential areas that have fewer opportunities for physical distancing. In addition, since this group primarily consists of forced immigrants, it is possible that older immigrants from these origins have moved to Sweden to live with relatives and are therefore more likely to be residing in multi-generational households [19]. This could result in higher levels of exposure compared to both Swedish-born individuals of the same age and immigrants from other origins [20].

To the best of our knowledge, this is the first study examining COVID-19 mortality by duration of residence; therefore, the opportunity for comparisons with previous studies is limited. However, our findings align with previous studies that, despite employing different designs, also support the exposure (as opposed to vulnerability) hypothesis. This includes studies controlling for diagnosed pre-existing conditions [7, 21], those based on morbidities prior to the pandemic [22], and those comparing intermarriage couples [6].

This finding has important public health impli-cations, as it suggests that measures implemented toward reducing exposure to the virus could have helped reduce inequalities by nativity. In this regard, a study conducted in Spain – a country that, unlike Sweden, implemented strong measures of disease control (e.g. prolonged lockdowns) – highlighted that inequalities in COVID-19 mortality were lower than in Sweden [23]. The extent to which these differences can be attributed to specific public health measures deserves further research.

Our findings extend beyond COVID-19 research. An international systematic review and meta-analysis [24, 25] confirmed the general mortality ad-vantage of immigrants, while a higher mortality for infectious diseases compared to the host-native population. Our study suggests that this pattern, particularly for airborne diseases, such as tuberculosis, may be explained by higher exposure in the country of origin. However, it also raises questions about whether complications from prior infectious diseases (which might not follow the pattern of duration of residence as other morbidities do) may have exacerbated COVID-19 mortality. A study revealed that most immigrant origins (predominantly from Africa and the Middle East) had higher hospitalisation rates for tuberculosis and HIV before the pandemic than those who were Swedish-born [22]. However, the study did not evaluate the contribution of these causes to the higher COVID-19 mortality among immigrants. Although such research is needed to reveal the complex interplay between exposure and susceptibility in relation to COVID-19 outcomes, the low prevalence of tuberculosis and HIV is unlikely to account for a significant portion of the observed inequalities in COVID-19 mortality among immigrants.

This study has important strengths. First, it used high-quality total population registers that provided a full representation of the mortality patterns by immigrants’ duration of residence. Second, given that free movement between countries was limited during the pandemic due to global restrictions (e.g. requiring pharmacy care records and vaccination certificates), it is unlikely that our results were affected by differential return migration (i.e. that people with relatively more or less time in Sweden decided to return to their home country). We also believe that our findings are robust since the results for all-cause mortality as well as for other causes of mortality before and during the pandemic were almost identical, minimising the possibility that our results were influenced by undertesting or misdiagnosis of COVID-19 mortality. Third, the indirect evaluation of susceptibility can be considered a strength as our results were not affected by differential levels of underdiagnosis, which are more common not only between immigrants and Swedish-born but also among immigrants by duration of residence.

This study also has limitations. Although we used high-quality data, duration of residence might be slightly underestimated since it is calculated from the date on which the residence permit is granted, not the date of arrival. In addition, we did not have information on underlying diseases, which could have been relevant to evaluate the influence of specific diagnoses.

In conclusion, Our study found that immigrants in Sweden had varying rates of COVID-19 deaths that did not depend on how long they had lived in the country. Unlike before the pandemic, when living in Sweden for a longer time typically meant a higher chance of death but still lower than that of Swedish-born people, COVID-19 deaths among immigrants did not follow this pattern. Moreover, populations from the origins that were most severely affected by the pandemic showed higher COVID-19 mortality hazards regardless of their duration of residence. In fact, higher hazards were found among those with shorter time in Sweden. As such, our study suggests that exposure (as opposed to susceptibility) played a greater role in explaining immigrants’ inequalities in relation to COVID-19 mortality, at least among the groups most affected by the pandemic.

## Supplemental Material

sj-docx-1-sjp-10.1177_14034948241244560 – Supplemental material for COVID-19 mortality among immigrants by duration of residence in Sweden: a population-based cohort study Supplemental material, sj-docx-1-sjp-10.1177_14034948241244560 for COVID-19 mortality among immigrants by duration of residence in Sweden: a population-based cohort study by Sol P. Juárez, Enrico Debiasi, Matthew Wallace, Sven Drefahl, Eleonora Mussino, Agneta Cederstrཡrm, Mikael Rostila and Siddartha Aradhya in Scandinavian Journal of Public Health
