# Supplementary material for: COVID-19 mortality among immigrants by duration of residence in Sweden: a population-based cohort study
Source: Scand J Public Health. 2024 Apr 10;52(3):370–8. doi: 10.1177/14034948241244560 (PMC11067384; doi:10.1177/14034948241244560)
Supplement: sj-docx-1-sjp-10.1177_14034948241244560 – Supplemental material for COVID-19 mortality among immigrants by duration of residence in Sweden: a population-based cohort study [file sj-docx-1-sjp-10.1177_14034948241244560.docx]

**Supplementary file**

**Table A1. Descriptive information for the pre-COVID period**

|  | **Swedish** | **Finland** | **Rest of Nordics** | **Western** | **Eastern Europe** | **Latin America** | **Africa** | **Middle East** | **South-East Asia** | **Rest of Asia** | **MISS** |
| --- | --- | --- | --- | --- | --- | --- | --- | --- | --- | --- | --- |
| N | 6792165 | 161129 | 94146 | 204488 | 328459 | 75640 | 197477 | 514795 | 141702 | 78825 | 148429 |
| Deaths | 394139 | 15692 | 6550 | 6200 | 9357 | 1198 | 1592 | 5512 | 845 | 534 | 3563 |
|  |  |  |  |  |  |  |  |  |  |  |  |
| **Sex** |  |  |  |  |  |  |  |  |  |  |  |
| Men | 50% | 40% | 50% | 54% | 48% | 48% | 54% | 57% | 40% | 46% | 53% |
| Women | 50% | 60% | 51% | 46% | 52% | 52% | 46% | 44% | 60% | 54% | 47% |
| **Age group** |  |  |  |  |  |  |  |  |  |  |  |
| 16-45 | 43% | 12% | 33% | 55% | 53% | 55% | 72% | 63% | 71% | 72% | 53% |
| 46-65 | 31% | 40% | 35% | 26% | 32% | 34% | 24% | 30% | 26% | 24% | 34% |
| 66-100 | 26% | 49% | 32% | 18% | 16% | 11% | 4% | 7% | 4% | 5% | 13% |
| **Age of arrival** |  |  |  |  |  |  |  |  |  |  |  |
| <18 |  | 38% | 27% | 18% | 21% | 37% | 21% | 22% | 27% | 15% | 28% |
| >=18 |  | 62% | 73% | 82% | 79% | 63% | 79% | 78% | 73% | 85% | 72% |
| **Duration of residence** |  |  |  |  |  |  |  |  |  |  |  |
| <15 |  | 7% | 31% | 57% | 47% | 30% | 71% | 59% | 58% | 72% | 34% |
| >=15 |  | 93% | 69% | 44% | 53% | 70% | 29% | 41% | 42% | 28% | 66% |
| **Civil status** |  |  |  |  |  |  |  |  |  |  |  |
| Not married | 43% | 25% | 29% | 40% | 27% | 39% | 32% | 27% | 36% | 31% | 32% |
| Married or Reg. Par. | 40% | 42% | 45% | 43% | 50% | 37% | 46% | 56% | 48% | 55% | 52% |
| Divorced | 11% | 21% | 18% | 12% | 19% | 22% | 19% | 14% | 14% | 12% | 13% |
| Widowed | 6% | 11% | 8% | 5% | 4% | 2% | 2% | 3% | 2% | 2% | 4% |
| **Income quintiles** |  |  |  |  |  |  |  |  |  |  |  |
| (Lowest) 1 | 16% | 23% | 37% | 34% | 30% | 27% | 35% | 38% | 36% | 41% | 29% |
| 2 | 20% | 22% | 16% | 17% | 22% | 22% | 28% | 27% | 21% | 19% | 19% |
| 3 | 21% | 21% | 15% | 16% | 19% | 20% | 18% | 16% | 18% | 15% | 18% |
| 4 | 22% | 18% | 15% | 15% | 16% | 16% | 12% | 11% | 13% | 12% | 17% |
| (Highest) 5 | 22% | 16% | 17% | 18% | 13% | 14% | 8% | 8% | 12% | 13% | 17% |
| **Educational attainment** |  |  |  |  |  |  |  |  |  |  |  |
| Primary | 18% | 29% | 19% | 12% | 16% | 16% | 33% | 30% | 25% | 14% | 12% |
| Secondary | 46% | 43% | 35% | 27% | 39% | 41% | 34% | 31% | 29% | 21% | 36% |
| Tertiary | 36% | 26% | 33% | 48% | 36% | 40% | 24% | 32% | 37% | 54% | 44% |
| MISS | 1% | 2% | 13% | 13% | 10% | 4% | 9% | 8% | 9% | 11% | 8% |

Note: Total may not always sum up to 100% due to rounding

Reg. Par.: Register partner; MISS.: Missing.

**Table A2. All-cause mortality before the pandemic (2015-2019) by age (group 18-65 and 66-100) and duration of residence. Unadjusted**

| **Duration of residence** | **All** | **<15 years** | **>15 years** | **All** | **<15 years** | **>15 years** |
| --- | --- | --- | --- | --- | --- | --- |
| **Age group** | **18-65** | **18-65** | **18-65** | **66-100** | **66-100** | **66-100** |
| **Sweden (ref)** | 1.00 | 1.00 | 1.00 | 1.00 | 1.00 | 1.00 |
| **Finland** | 1.35^***^ | 0.78 | 1.38^***^ | 1.15^***^ | 1.21 | 1.15^***^ |
|  | [1.29,1.41] | [0.57,1.07] | [1.32,1.44] | [1.13,1.17] | [0.99,1.48] | [1.13,1.17] |
| **Rest of Nordics** | 1.24^***^ | 1.33^***^ | 1.22^***^ | 1.10^***^ | 1.34^***^ | 1.08^***^ |
|  | [1.15,1.33] | [1.15,1.53] | [1.12,1.33] | [1.07,1.13] | [1.22,1.46] | [1.05,1.11] |
| **Western** | 0.81^***^ | 0.65^***^ | 0.94 | 0.91^***^ | 0.94 | 0.90^***^ |
|  | [0.75,0.87] | [0.58,0.73] | [0.86,1.02] | [0.88,0.93] | [0.85,1.04] | [0.88,0.93] |
| **Eastern Europe** | 0.94^**^ | 0.85^***^ | 0.99 | 1.00 | 1.03 | 1.00 |
|  | [0.90,0.98] | [0.78,0.92] | [0.94,1.04] | [0.97,1.02] | [0.93,1.14] | [0.97,1.02] |
| **Latin America** | 0.66^***^ | 0.51^***^ | 0.70^***^ | 0.81^***^ | 0.64 | 0.82^***^ |
|  | [0.60,0.74] | [0.39,0.67] | [0.63,0.78] | [0.76,0.87] | [0.41,1.01] | [0.76,0.88] |
| **Africa** | 1.01 | 0.91 | 1.10^*^ | 0.84^***^ | 0.65^***^ | 0.90^*^ |
|  | [0.94,1.07] | [0.83,1.00] | [1.01,1.20] | [0.77,0.90] | [0.55,0.78] | [0.83,0.98] |
| **Middle East** | 0.72^***^ | 0.64^***^ | 0.78^***^ | 0.85^***^ | 0.82^***^ | 0.86^***^ |
|  | [0.69,0.76] | [0.60,0.69] | [0.74,0.83] | [0.82,0.88] | [0.76,0.88] | [0.82,0.89] |
| **South-East Asia** | 0.69^***^ | 0.54^***^ | 0.79^***^ | 0.87^**^ | 0.60 | 0.88^*^ |
|  | [0.63,0.76] | [0.46,0.64] | [0.71,0.89] | [0.79,0.96] | [0.35,1.04] | [0.80,0.98] |
| **Rest of Asia** | 0.67^***^ | 0.58^***^ | 0.78^**^ | 0.76^***^ | 1.05 | 0.73^***^ |
|  | [0.59,0.76] | [0.48,0.71] | [0.65,0.94] | [0.68,0.85] | [0.78,1.41] | [0.64,0.82] |
| **MISS** | 0.94 | 0.80^**^ | 0.98 | 1.05^*^ | 0.83^*^ | 1.06^**^ |
|  | [0.88,1.00] | [0.68,0.92] | [0.91,1.06] | [1.01,1.09] | [0.69,1.00] | [1.02,1.10] |
| **N of failures** | 57286 | 49630 | 54488 | 387896 | 349683 | 385520 |
| **Time at risk (person-years)** | 30250139.74 | 27238306.78 | 26411140.53 | 9569420.84 | 8507144.65 | 9469900.84 |

Since the Swedish population is the reference group, the failures are included in both categories of duration of residence.

^*^ *p* < 0.05, ^**^ *p* < 0.01, ^***^ *p* < 0.001

MISS.: Missing.

**Table A3. All-cause mortality before the pandemic (2015-2019) by age (group 18-65 and 66-100) and duration of residence. Adjusted**

| **Duration of residence** | **All** | **<15 years** | **>15 years** | **All** | **<15 years** | **>15 years** |
| --- | --- | --- | --- | --- | --- | --- |
| **Age group** | **18-65** | **18-65** | **18-65** | **66-100** | **66-100** | **66-100** |
| **Sweden (ref)** | 1.00 | 1.00 | 1.00 | 1.00 | 1.00 | 1.00 |
| **Finland** | 1.08^***^ | 0.60^**^ | 1.10^***^ | 1.12^***^ | 1.12 | 1.12^***^ |
|  | [1.03,1.13] | [0.44,0.82] | [1.05,1.15] | [1.10,1.14] | [0.92,1.37] | [1.10,1.14] |
| **Rest of Nordics** | 0.92^*^ | 0.63^***^ | 1.00 | 1.06^***^ | 1.09 | 1.06^***^ |
|  | [0.85,0.99] | [0.55,0.73] | [0.92,1.09] | [1.04,1.09] | [0.99,1.20] | [1.03,1.09] |
| **Western** | 0.60^***^ | 0.39^***^ | 0.77^***^ | 0.91^***^ | 0.84^**^ | 0.91^***^ |
|  | [0.56,0.64] | [0.35,0.44] | [0.71,0.84] | [0.89,0.94] | [0.75,0.93] | [0.89,0.94] |
| **Eastern Europe** | 0.72^***^ | 0.52^***^ | 0.81^***^ | 0.98 | 0.94 | 0.98 |
|  | [0.69,0.75] | [0.48,0.56] | [0.76,0.85] | [0.96,1.01] | [0.84,1.04] | [0.96,1.01] |
| **Latin America** | 0.50^***^ | 0.33^***^ | 0.54^***^ | 0.77^***^ | 0.56^*^ | 0.77^***^ |
|  | [0.45,0.56] | [0.25,0.43] | [0.48,0.60] | [0.72,0.82] | [0.36,0.89] | [0.72,0.83] |
| **Africa** | 0.65^***^ | 0.52^***^ | 0.77^***^ | 0.77^***^ | 0.57^***^ | 0.83^***^ |
|  | [0.61,0.70] | [0.47,0.57] | [0.70,0.84] | [0.71,0.83] | [0.48,0.68] | [0.76,0.90] |
| **Middle East** | 0.47^***^ | 0.37^***^ | 0.54^***^ | 0.80^***^ | 0.73^***^ | 0.81^***^ |
|  | [0.45,0.50] | [0.34,0.40] | [0.51,0.57] | [0.77,0.83] | [0.68,0.79] | [0.77,0.84] |
| **South-East Asia** | 0.48^***^ | 0.32^***^ | 0.59^***^ | 0.84^***^ | 0.54^*^ | 0.85^**^ |
|  | [0.44,0.53] | [0.27,0.37] | [0.52,0.66] | [0.76,0.93] | [0.31,0.94] | [0.77,0.94] |
| **Rest of Asia** | 0.50^***^ | 0.37^***^ | 0.66^***^ | 0.75^***^ | 0.97 | 0.72^***^ |
|  | [0.44,0.57] | [0.30,0.44] | [0.55,0.79] | [0.67,0.84] | [0.72,1.30] | [0.64,0.81] |
| **MISS** | 0.78^***^ | 0.51^***^ | 0.87^***^ | 1.04 | 0.72^***^ | 1.05^*^ |
|  | [0.73,0.84] | [0.44,0.59] | [0.80,0.93] | [1.00,1.08] | [0.60,0.87] | [1.01,1.10] |
| **N of failures** | 57286 | 49630 | 54488 | 387896 | 349683 | 385520 |
| **Time at risk (person-years)** | 30250139.74 | 27238306.78 | 26411140.53 | 9569420.84 | 8507144.65 | 9469900.84 |

Adjusted for age, sex, education, civil status, income, and county of residence fixed effect.

Since the Swedish population is the reference group, the failures are included in both categories of duration of residence.

^*^ *p* < 0.05, ^**^ *p* < 0.01, ^***^ *p* < 0.001

MISS.: Missing.

**Table A4. All-cause mortality before the pandemic (2015-2019) by sex and duration of residence for the working-age population. Adjusted**

| **Sex** |  |  |  |  | **Men** |  |  |  |
| --- | --- | --- | --- | --- | --- | --- | --- | --- |
| **Duration of residence** | **<5** | **5-9** | **10-14** | **15-19** | **20-24** | **25-30** | **30-34** | **35+** |
| **Sweden(ref)** | 1.00 | 1.00 | 1.00 | 1.00 | 1.00 | 1.00 | 1.00 | 1.00 |
| **Migrants** | 0.38^***^ | 0.47^***^ | 0.51^***^ | 0.62^***^ | 0.79^***^ | 0.75^***^ | 0.68^***^ | 0.91^***^ |
|  | [0.35,0.41] | [0.43,0.51] | [0.47,0.56] | [0.56,0.69] | [0.72,0.86] | [0.70,0.80] | [0.62,0.74] | [0.87,0.96] |
| **NA** | 0.33^***^ | 0.46^***^ | 0.70^*^ | 1.03 | 1.01 | 0.45^***^ | 1.18 | 0.93 |
|  | [0.24,0.47] | [0.32,0.64] | [0.52,0.94] | [0.81,1.32] | [0.88,1.15] | [0.33,0.61] | [0.84,1.66] | [0.74,1.15] |
| **N of failures** | 29499 | 29406 | 29310 | 29219 | 29561 | 29647 | 29358 | 30872 |
| **Time at risk** | 12884427.34 | 12616226.36 | 12408931.09 | 12242353.57 | 12284803.70 | 12295970.57 | 12147219.07 | 12322074.78 |
| **Sex** |  |  |  |  | **Women** |  |  |  |
| **Duration of residence** | **<5** | **5-9** | **10-14** | **15-19** | **20-24** | **25-30** | **30-34** | **35+** |
| **Native (ref)** | 1.00 | 1.00 | 1.00 | 1.00 | 1.00 | 1.00 | 1.00 | 1.00 |
| **Migrants** | 0.30^***^ | 0.36^***^ | 0.41^***^ | 0.54^***^ | 0.61^***^ | 0.60^***^ | 0.61^***^ | 0.84^***^ |
|  | [0.26,0.33] | [0.32,0.41] | [0.36,0.47] | [0.47,0.61] | [0.54,0.68] | [0.54,0.66] | [0.54,0.68] | [0.79,0.89] |
|  | 0.25^***^ | 0.61^*^ | 0.70 | 0.75 | 0.84^*^ | 0.18^***^ | 1.00 | 1.21 |
| **MISS** | [0.15,0.43] | [0.40,0.92] | [0.47,1.04] | [0.51,1.08] | [0.71,1.00] | [0.10,0.33] | [0.61,1.64] | [0.98,1.51] |
|  | 0.30^***^ | 0.36^***^ | 0.41^***^ | 0.54^***^ | 0.61^***^ | 0.60^***^ | 0.61^***^ | 0.84^***^ |
| **N of failures** | 18373 | 18370 | 18336 | 18314 | 18489 | 18491 | 18355 | 19510 |
| **Time at risk**  **(person-years)** | 12188213.61 | 12049729.51 | 11889394.03 | 11745657.25 | 11773582.36 | 11753186.92 | 11605444.65 | 11838077.98 |

Models adjusted for age, education, income, civil status and county of residence fixed effect

Since the Swedish population is the reference group, the failures are included in both categories of duration of residence.

^*^ *p* < 0.05, ^**^ *p* < 0.01, ^***^ *p* < 0.001

MISS.: Missing.

**Table A5. All-cause mortality before the pandemic (2015-2019) by sex and duration of residence for the retirement age population. Adjusted**

|  |  |  |  |  | **Men** |  |  |  |
| --- | --- | --- | --- | --- | --- | --- | --- | --- |
|  | **<5** | **5-9** | **10-14** | **15-19** | **20-24** | **25-30** | **30-34** | **35+** |
| **Sweden (ref)** | 1.00 | 1.00 | 1.00 | 1.00 | 1.00 | 1.00 | 1.00 | 1.00 |
| **Migrants** | 0.79^***^ | 0.76^***^ | 0.90^*^ | 0.82^***^ | 0.91^*^ | 0.86^***^ | 0.82^***^ | 1.03^**^ |
|  | [0.70,0.88] | [0.69,0.85] | [0.81,0.99] | [0.74,0.91] | [0.83,0.99] | [0.80,0.92] | [0.76,0.88] | [1.01,1.05] |
| **NA** | 0.39^**^ | 0.60^*^ | 0.99 | 1.42^**^ | 1.30^***^ | 0.36^***^ | 1.04 | 0.88^**^ |
|  | [0.21,0.72] | [0.39,0.91] | [0.73,1.36] | [1.14,1.77] | [1.19,1.42] | [0.27,0.49] | [0.74,1.46] | [0.81,0.96] |
| **N of failures** | 165824 | 165893 | 166005 | 165990 | 166630 | 166430 | 166154 | 179952 |
| **Time at risk**  **(person-years)** | 3919105.03 | 3920963.14 | 3920864.06 | 3919089.59 | 3931954.96 | 3939808.85 | 3931554.26 | 4267579.60 |
|  |  |  |  |  | **Women** |  |  |  |
|  | **<5** | **5-9** | **10-14** | **15-19** | **20-24** | **25-30** | **30-34** | **35+** |
| **Native (ref)** | 1.00 | 1.00 | 1.00 | 1.00 | 1.00 | 1.00 | 1.00 | 1.00 |
| **Migrants** | 0.75^***^ | 0.87^*^ | 0.81^***^ | 0.94 | 0.91^**^ | 0.81^***^ | 0.79^***^ | 1.03^**^ |
|  | [0.66,0.85] | [0.78,0.97] | [0.73,0.90] | [0.85,1.04] | [0.84,0.98] | [0.76,0.87] | [0.73,0.85] | [1.01,1.04] |
| **MISS** | 0.76 | 0.25^**^ | 0.93 | 1.59^***^ | 1.39^***^ | 0.37^***^ | 0.66 | 0.87^**^ |
|  | [0.43,1.35] | [0.09,0.66] | [0.62,1.40] | [1.28,1.97] | [1.28,1.51] | [0.27,0.50] | [0.38,1.14] | [0.79,0.96] |
| **N of failures** | 182111 | 182233 | 182231 | 182368 | 183380 | 182898 | 182549 | 198397 |
| **Time at risk**  **(person-years)** | 4518103.57 | 4521500.52 | 4521857.64 | 4521796.80 | 4542630.86 | 4544870.04 | 4535091.89 | 4966022.64 |

Models adjusted for age, education, income, civil status, and county of residence fixed effect

Since the Swedish population is the reference group, the failures are included in both categories of duration of residence.

^*^ *p* < 0.05, ^**^ *p* < 0.01, ^***^ *p* < 0.001

MISS.: Missing.

**Table A6. COVID-19 mortality by age (group 18-65 and 66-100) and duration of residence. Unadjusted**

| **Duration of residence** | **All** | **<15 years** | **>15 years** | **All** | **<15 years** | **>15 years** |
| --- | --- | --- | --- | --- | --- | --- |
| **Age group** | **18-65** | **18-65** | **18-65** | **66-100** | **66-100** | **66-100** |
| **Sweden (ref)** | 1.00 | 1.00 | 1.00 | 1.00 | 1.00 | 1.00 |
| **Finland** | 1.53^**^ | 0.64 | 1.59^**^ | 1.52^***^ | 0.88 | 1.52^***^ |
|  | [1.13,2.07] | [0.09,4.60] | [1.17,2.16] | [1.42,1.63] | [0.28,2.71] | [1.42,1.63] |
| **Rest of Nordics** | 0.69 | 0.71 | 0.64 | 1.02 | 0.97 | 1.02 |
|  | [0.37,1.28] | [0.23,2.22] | [0.30,1.35] | [0.89,1.16] | [0.54,1.75] | [0.89,1.16] |
| **Western** | 0.92 | 0.44^*^ | 1.31 | 1.15^*^ | 0.86 | 1.17^**^ |
|  | [0.60,1.39] | [0.20,0.99] | [0.81,2.13] | [1.03,1.29] | [0.51,1.46] | [1.04,1.31] |
| **Eastern Europe** | 1.54^***^ | 1.00 | 1.81^***^ | 1.55^***^ | 1.06 | 1.58^***^ |
|  | [1.21,1.96] | [0.62,1.60] | [1.37,2.38] | [1.42,1.69] | [0.66,1.70] | [1.44,1.72] |
| **Latin America** | 2.00^**^ | 0.84 | 2.26^***^ | 1.88^***^ | 1.64 | 1.89^***^ |
|  | [1.31,3.06] | [0.21,3.36] | [1.45,3.52] | [1.52,2.32] | [0.41,6.54] | [1.52,2.34] |
| **Africa** | 3.56^***^ | 4.62^***^ | 2.35^***^ | 2.99^***^ | 4.51^***^ | 2.53^***^ |
|  | [2.80,4.52] | [3.41,6.28] | [1.61,3.43] | [2.50,3.59] | [3.34,6.09] | [2.01,3.17] |
| **Middle East** | 2.46^***^ | 2.15^***^ | 2.59^***^ | 3.19^***^ | 3.92^***^ | 2.97^***^ |
|  | [2.08,2.92] | [1.64,2.81] | [2.11,3.18] | [2.94,3.46] | [3.38,4.54] | [2.70,3.27] |
| **South-East Asia** | 1.98^***^ | 0.83 | 2.71^***^ | 1.94^***^ | 0.94 | 1.99^***^ |
|  | [1.38,2.84] | [0.37,1.88] | [1.82,4.04] | [1.45,2.60] | [0.13,6.69] | [1.48,2.68] |
| **Rest of Asia** | 2.69^***^ | 1.99^*^ | 3.18^***^ | 2.18^***^ | 2.26 | 2.18^***^ |
|  | [1.74,4.16] | [1.02,3.87] | [1.80,5.62] | [1.60,2.98] | [0.85,6.02] | [1.57,3.02] |
| **MISS** | 1.67^**^ | 1.31 | 1.75^**^ | 1.55^***^ | 0.55 | 1.59^***^ |
|  | [1.20,2.34] | [0.65,2.64] | [1.20,2.55] | [1.34,1.79] | [0.18,1.72] | [1.38,1.84] |
| **N of failures** | 1354 | 1018 | 1195 | 17209 | 14507 | 16931 |
| **Time at risk**  **(person-years)** | 5717711.49 | 5127482.35 | 4875440.28 | 1922396.17 | 1695517.84 | 1900553.64 |

Since the Swedish population is the reference group, the failures are included in both categories of duration of residence.

^*^ *p* < 0.05, ^**^ *p* < 0.01, ^***^ *p* < 0.001

MISS.: Missing.

**Table A7. COVID-19 mortality by age (group 18-65 and 66-100) and duration of residence. Adjusted**

| **Duration of residence** | **All** | **<15 years** | **>15 years** | **All** | **<15 years** | **>15 years** |
| --- | --- | --- | --- | --- | --- | --- |
| **Age group** | **18-65** | **18-65** | **18-65** | **66-100** | **66-100** | **66-100** |
| **Sweden (ref)** | 1.00 | 1.00 | 1.00 | 1.00 | 1.00 | 1.00 |
| **Finland** | 1.22 | 0.57 | 1.26 | 1.42^***^ | 0.85 | 1.42^***^ |
|  | [0.90,1.65] | [0.08,4.08] | [0.92,1.72] | [1.32,1.52] | [0.27,2.65] | [1.32,1.53] |
| **Rest of Nordics** | 0.68 | 0.54 | 0.62 | 1.05 | 1.20 | 1.05 |
|  | [0.36,1.27] | [0.17,1.71] | [0.29,1.31] | [0.92,1.20] | [0.65,2.21] | [0.92,1.20] |
| **Western** | 0.68 | 0.26^**^ | 1.04 | 1.00 | 0.86 | 1.01 |
|  | [0.44,1.03] | [0.11,0.59] | [0.64,1.68] | [0.89,1.11] | [0.50,1.47] | [0.90,1.13] |
| **Eastern Europe** | 1.18 | 0.53^*^ | 1.61^***^ | 1.34^***^ | 0.96 | 1.36^***^ |
|  | [0.92,1.52] | [0.32,0.87] | [1.21,2.13] | [1.22,1.46] | [0.59,1.58] | [1.24,1.49] |
| **Latin America** | 1.37 | 0.50 | 1.46 | 1.32^*^ | 1.25 | 1.33^*^ |
|  | [0.90,2.11] | [0.12,2.00] | [0.93,2.29] | [1.07,1.64] | [0.31,5.00] | [1.07,1.65] |
| **Africa** | 2.18^***^ | 2.46^***^ | 1.49^*^ | 2.06^***^ | 3.94^***^ | 1.66^***^ |
|  | [1.70,2.80] | [1.78,3.38] | [1.01,2.19] | [1.71,2.48] | [2.85,5.44] | [1.32,2.09] |
| **Middle East** | 1.55^***^ | 1.20 | 1.65^***^ | 2.30^***^ | 3.27^***^ | 2.12^***^ |
|  | [1.29,1.86] | [0.90,1.60] | [1.32,2.05] | [2.10,2.52] | [2.70,3.97] | [1.91,2.34] |
| **South-East Asia** | 1.50^*^ | 0.51 | 2.15^***^ | 1.59^**^ | 0.80 | 1.64^**^ |
|  | [1.04,2.16] | [0.23,1.16] | [1.43,3.22] | [1.18,2.13] | [0.11,5.71] | [1.22,2.21] |
| **Rest of Asia** | 1.84^**^ | 1.09 | 2.46^**^ | 1.71^***^ | 1.91 | 1.71^**^ |
|  | [1.18,2.87] | [0.55,2.15] | [1.38,4.37] | [1.25,2.33] | [0.71,5.12] | [1.23,2.37] |
| **MISS** | 1.40 | 0.81 | 1.56^*^ | 1.37^***^ | 0.48 | 1.43^***^ |
|  | [1.00,1.97] | [0.39,1.65] | [1.07,2.28] | [1.18,1.59] | [0.15,1.50] | [1.23,1.66] |
| **N of failures** | 1354 | 1018 | 1195 | 17209 | 14507 | 16931 |
| **Time at risk**  **(person-years)** | 5717711.49 | 5127482.35 | 4875440.28 | 1922396.17 | 1695517.84 | 1900553.64 |

Models adjusted for age, sex, education, income, civil status, and county of residence fixed effect

Since the Swedish population is the reference group, the failures are included in both categories of duration of residence.

^*^ *p* < 0.05, ^**^ *p* < 0.01, ^***^ *p* < 0.001

MISS.: Missing.

**Table A8. All other causes of deaths (all excluding COVID-19) during pandemic by age (group 18-65 and 66-100) by duration of residence. Unadjusted**

| **Duration of residence** | **All** | **<15 years** | **>15 years** | **All** | **<15 years** | **>15 years** |
| --- | --- | --- | --- | --- | --- | --- |
| **Age group** | **18-65** | **18-65** | **18-65** | **66-100** | **66-100** | **66-100** |
| **Sweden (ref)** | 1.00 | 1.00 | 1.00 | 1.00 | 1.00 | 1.00 |
| **Finland** | 1.23^***^ | 0.68 | 1.27^***^ | 1.15^***^ | 1.33 | 1.15^***^ |
|  | [1.14,1.33] | [0.44,1.05] | [1.18,1.37] | [1.12,1.18] | [1.00,1.77] | [1.12,1.18] |
| **Rest of Nordics** | 0.96 | 0.57^***^ | 1.09 | 1.04^*^ | 0.95 | 1.05^*^ |
|  | [0.85,1.08] | [0.42,0.76] | [0.96,1.24] | [1.00,1.08] | [0.79,1.13] | [1.00,1.09] |
| **Western** | 0.72^***^ | 0.55^***^ | 0.88^*^ | 0.92^***^ | 0.83^*^ | 0.93^***^ |
|  | [0.65,0.80] | [0.47,0.65] | [0.77,1.00] | [0.89,0.96] | [0.71,0.97] | [0.89,0.97] |
| **Eastern Europe** | 0.96 | 0.90^*^ | 1.00 | 1.04^*^ | 0.92 | 1.05^**^ |
|  | [0.90,1.03] | [0.81,1.00] | [0.92,1.08] | [1.01,1.07] | [0.79,1.07] | [1.01,1.08] |
| **South America** | 0.69^***^ | 0.33^***^ | 0.79^**^ | 0.83^***^ | 0.47^*^ | 0.84^***^ |
|  | [0.60,0.81] | [0.21,0.53] | [0.67,0.92] | [0.76,0.91] | [0.23,0.99] | [0.77,0.92] |
| **Africa** | 1.03 | 0.89 | 1.15^*^ | 0.89^*^ | 0.69^***^ | 0.97 |
|  | [0.94,1.12] | [0.78,1.01] | [1.03,1.30] | [0.81,0.98] | [0.55,0.85] | [0.87,1.08] |
| **Middle East** | 0.81^***^ | 0.76^***^ | 0.85^***^ | 0.92^***^ | 1.00 | 0.90^***^ |
|  | [0.77,0.86] | [0.70,0.83] | [0.79,0.91] | [0.89,0.97] | [0.92,1.09] | [0.86,0.95] |
| **South-east Asia** | 0.72^***^ | 0.52^***^ | 0.87 | 0.93 | 0.50 | 0.95 |
|  | [0.64,0.81] | [0.42,0.63] | [0.75,1.01] | [0.82,1.05] | [0.24,1.04] | [0.84,1.08] |
| **Rest of Asia** | 0.59^***^ | 0.46^***^ | 0.75^*^ | 0.72^***^ | 0.70 | 0.72^***^ |
|  | [0.49,0.71] | [0.35,0.60] | [0.58,0.96] | [0.62,0.84] | [0.42,1.16] | [0.61,0.86] |
| **MISS** | 0.86^**^ | 0.66^***^ | 0.93 | 1.06^*^ | 0.79 | 1.08^**^ |
|  | [0.78,0.95] | [0.53,0.81] | [0.83,1.04] | [1.01,1.12] | [0.60,1.04] | [1.02,1.14] |
| **N of failures** | 6249738 | 5607150 | 5310427 | 2045771 | 1803984 | 2022035 |
| **Time at risk**  **(person-years)** | 24733 | 21212 | 23127 | 176983 | 157513 | 175787 |

Since the Swedish population is the reference group, the failures are included in both categories of duration of residence.

^*^ *p* < 0.05, ^**^ *p* < 0.01, ^***^ *p* < 0.001

MISS.: Missing.

**Table A9. All other causes of deaths (all excluding COVID-19) during pandemic by age (group 18-65 and 66-100) by duration of residence. Adjusted**

| **Duration of residence** | **All** | **<15 years** | **>15 years** | **All** | **<15 years** | **>15 years** |
| --- | --- | --- | --- | --- | --- | --- |
| **Age group** | **18-65** | **18-65** | **18-65** | **66-100** | **66-100** | **66-100** |
| **Sweden (ref)** | 1.00 | 1.00 | 1.00 | 1.00 | 1.00 | 1.00 |
| **Finland** | 1.06 | 0.46^***^ | 1.10^*^ | 1.13^***^ | 0.31^***^ | 1.13^***^ |
|  | [0.98,1.14] | [0.30,0.71] | [1.02,1.19] | [1.10,1.16] | [0.23,0.42] | [1.11,1.16] |
| **Rest of Nordics** | 0.69^***^ | 0.23^***^ | 0.85^*^ | 0.78^***^ | 0.13^***^ | 0.85^***^ |
|  | [0.61,0.78] | [0.17,0.31] | [0.75,0.97] | [0.75,0.81] | [0.11,0.16] | [0.82,0.88] |
| **Western** | 0.47^***^ | 0.24^***^ | 0.73^***^ | 0.70^***^ | 0.13^***^ | 0.78^***^ |
|  | [0.43,0.53] | [0.21,0.28] | [0.65,0.83] | [0.67,0.73] | [0.11,0.15] | [0.75,0.81] |
| **Eastern Europe** | 0.66^***^ | 0.38^***^ | 0.83^***^ | 0.74^***^ | 0.14^***^ | 0.78^***^ |
|  | [0.62,0.71] | [0.34,0.43] | [0.77,0.90] | [0.71,0.76] | [0.12,0.16] | [0.75,0.80] |
| **Latin America** | 0.52^***^ | 0.18^***^ | 0.60^***^ | 0.56^***^ | 0.08^***^ | 0.56^***^ |
|  | [0.45,0.61] | [0.11,0.28] | [0.51,0.70] | [0.51,0.62] | [0.04,0.17] | [0.51,0.62] |
| **Africa** | 0.66^***^ | 0.44^***^ | 0.83^**^ | 0.38^***^ | 0.11^***^ | 0.47^***^ |
|  | [0.60,0.72] | [0.39,0.51] | [0.74,0.94] | [0.35,0.42] | [0.09,0.13] | [0.42,0.53] |
| **Middle East** | 0.53^***^ | 0.39^***^ | 0.59^***^ | 0.39^***^ | 0.15^***^ | 0.42^***^ |
|  | [0.50,0.56] | [0.36,0.43] | [0.55,0.64] | [0.37,0.41] | [0.14,0.17] | [0.39,0.44] |
| **South-East Asia** | 0.49^***^ | 0.25^***^ | 0.68^***^ | 0.60^***^ | 0.09^***^ | 0.61^***^ |
|  | [0.43,0.55] | [0.21,0.31] | [0.58,0.78] | [0.53,0.68] | [0.04,0.19] | [0.54,0.70] |
| **Rest of Asia** | 0.40^***^ | 0.22^***^ | 0.65^***^ | 0.41^***^ | 0.13^***^ | 0.42^***^ |
|  | [0.33,0.48] | [0.17,0.29] | [0.51,0.84] | [0.35,0.48] | [0.08,0.21] | [0.36,0.50] |
| **MISS** | 0.67^***^ | 0.31^***^ | 0.81^***^ | 0.58^***^ | 0.12^***^ | 0.57^***^ |
|  | [0.61,0.74] | [0.25,0.39] | [0.73,0.91] | [0.55,0.61] | [0.09,0.16] | [0.54,0.60] |
| **N of failures** | 24733 | 21212 | 23127 | 176983 | 157513 | 175787 |
| **Time at risk**  **(person-years)** | 5717711.49 | 5127482.35 | 4875440.28 | 1922396.17 | 1695517.84 | 1900553.64 |

Adjusted for age, sex, education, civil status, income, and county of residence fixed effect.

Since the Swedish population is the reference group, the failures are included in both categories of duration of residence.

^*^ *p* < 0.05, ^**^ *p* < 0.01, ^***^ *p* < 0.001

MISS.: Missing.

**Table A10. Sensitivity analysis: COVID-19 mortality by duration of residence. Only among immigrants who arrived to Sweden as adults (18+)**

| **Duration of residence** | **All** | **<15 years** | **>15 years** | **All** | **<15 years** | **>15 years** |
| --- | --- | --- | --- | --- | --- | --- |
| **Age group** | **18-65** | **18-65** | **18-65** | **66-100** | **66-100** | **66-100** |
| **Sweden (ref)** | 1.00 | 1.00 | 1.00 | 1.00 | 1.00 | 1.00 |
| **Finland** | 1.43 | 0.59 | 1.51 | 1.42^***^ | 0.85 | 1.43^***^ |
|  | [0.93,2.19] | [0.08,4.21] | [0.98,2.35] | [1.32,1.54] | [0.27,2.65] | [1.32,1.54] |
| **Rest of Nordics** | 0.73 | 0.56 | 0.67 | 1.05 | 1.20 | 1.05 |
|  | [0.36,1.48] | [0.18,1.78] | [0.28,1.63] | [0.91,1.20] | [0.65,2.21] | [0.91,1.20] |
| **Western** | 0.55^*^ | 0.27^**^ | 0.86 | 0.99 | 0.86 | 1.01 |
|  | [0.33,0.90] | [0.12,0.62] | [0.46,1.61] | [0.88,1.12] | [0.50,1.47] | [0.89,1.14] |
| **Eastern Europe** | 1.11 | 0.52^*^ | 1.63^**^ | 1.34^***^ | 0.96 | 1.37^***^ |
|  | [0.84,1.47] | [0.31,0.86] | [1.19,2.24] | [1.23,1.47] | [0.59,1.58] | [1.25,1.51] |
| **Latin America** | 1.20 | 0.51 | 1.30 | 1.32^*^ | 1.25 | 1.33^*^ |
|  | [0.73,1.98] | [0.13,2.07] | [0.76,2.23] | [1.07,1.64] | [0.31,5.00] | [1.07,1.65] |
| **Africa** | 2.26^***^ | 2.54^***^ | 1.57^*^ | 2.07^***^ | 3.94^***^ | 1.68^***^ |
|  | [1.75,2.91] | [1.84,3.51] | [1.05,2.34] | [1.72,2.50] | [2.85,5.44] | [1.33,2.11] |
| **Middle East** | 1.53^***^ | 1.21 | 1.67^***^ | 2.31^***^ | 3.27^***^ | 2.13^***^ |
|  | [1.26,1.86] | [0.90,1.62] | [1.32,2.11] | [2.11,2.53] | [2.70,3.97] | [1.92,2.36] |
| **South-East Asia** | 1.22 | 0.54 | 1.77^*^ | 1.60^**^ | 0.80 | 1.65^***^ |
|  | [0.80,1.86] | [0.24,1.21] | [1.09,2.89] | [1.19,2.15] | [0.11,5.71] | [1.23,2.23] |
| **Rest of Asia** | 1.75^*^ | 1.13 | 2.32^**^ | 1.73^***^ | 1.91 | 1.73^**^ |
|  | [1.09,2.79] | [0.57,2.23] | [1.24,4.36] | [1.27,2.36] | [0.71,5.12] | [1.25,2.41] |
| **MISS** | 1.48^*^ | 0.83 | 1.76^**^ | 1.41^***^ | 0.48 | 1.47^***^ |
|  | [1.04,2.10] | [0.41,1.70] | [1.18,2.61] | [1.21,1.63] | [0.15,1.50] | [1.27,1.71] |
| **N of failures** | 1275 | 1014 | 1120 | 17009 | 14507 | 16731 |
| **Time at risk**  **(person-years)** | 5340216.15 | 5017272.66 | 4608154.63 | 1891781.85 | 1695517.84 | 1869939.31 |

Adjusted for age, sex, education, civil status, income, and county of residence fixed effect.

Since the Swedish population is the reference group, the failures are included in both categories of duration of residence.

^*^ *p* < 0.05, ^**^ *p* < 0.01, ^***^ *p* < 0.001

MISS.: Missing.
